# Nineteenth Annual Report of the State Board of Agriculture of the State of Michigan

**Published:** 1882-04

**Authors:** 


NINETEENTH ANNUAL REPORT OF THE STATE BOARD OF AGRICULTURE OF THE STATE OF MICHIGAN, for the year ending August 31st, 1880. ROBERT G. BAIRD, Secretary. Lansing: 1880. 8vo, pp. 496. Cuts.

This interesting report contains much valuable matter not only to farmers, but to breeders of stock. The report of the cattle commission gives a full and detailed account of the Texas cattle-disease, pleuro-pneumonia and glanders. It also recommends certain rules to prevent the spread of the last-mentioned disease. A large portion of the volume is devoted to the proceedings of the farmers' institutes, containing discussions on Jersey cattle, short-horns, management of sheep, insect pests and many other things important to farmers.

